# Data on beetle-killed and surviving lodgepole pine (*Pinus contorta*) radial growth from the Beaverhead-Deerlodge National Forest, MT prior to a severe mountain pine beetle outbreak

**DOI:** 10.1016/j.dib.2018.08.019

**Published:** 2018-08-14

**Authors:** L. Annie Cooper, Charlotte C. Reed, Ashley P. Ballantyne

**Affiliations:** University of Montana, Missoula, MT, United States

## Abstract

This article contains measurements of raw radial growth, distance to pith, and calculated basal area increments (BAI) from 444 5-mm increment cores (237 trees) collected in July 2016 from the Beaverhead-Deerlodge National Forest, MT. These data were used for the study presented in “Mountain pine beetle attack faster growing lodgepole pine at low elevations in western Montana, USA” [1]. Plot locations where increment cores were taken as well as code to calculate BAI are also included. Cores were collected from lodgepole pine (*Pinus contorta*) trees that were killed during a recent bark beetle outbreak (220 cores; 117 trees) as well as trees that survived the outbreak (210 cores; 113 trees) in twelve stands spanning north and south aspects and three elevational bands along a 600-m gradient. 14 additional cores were collected from 7 strip-attacked trees. Increment cores were prepared and measured using standard dendrochronological techniques, “An Introduction to Tree-Ring Dating” [2]. Master chronologies for each aspect-elevation combination were created using approximately ten cores from surviving trees at each location. Cores were cross-dated, then scanned at 2400 dpi. Annual ring widths were measured using CooRecorder 7.7, “Cybis Electronic, CDendro and CooRecorder V.7.7” [3], and final chronologies were quantitatively validated in COFECHA, “Computer-assisted quality control in tree-ring dating and measurement, Tree-Ring Society” [4].

**Specifications Table**

**Table:** 

Subject area	*Ecology*
More specific subject area	*Dendrochronology*
Type of data	*Table*
How data were acquired	*Field measurements using a 5-mm increment borer.*
Data format	*Raw; analyzed*
Experimental factors	*Two cores were collected per tree. Increment cores were prepared using standard dendrochronological techniques as in*[2].
Experimental features	*Data were collected from north/south aspects and three elevational bands, and from both beetle-killed and surviving trees.*
Data source location	*Beaverhead-Deerlodge National Forest, MT*
Data accessibility	*With this article*
Related research article	Cooper et al. [1]

**Value of the data**

● These data are unique in containing growth information for both beetle-killed and surviving trees from a common species in western North America.
● These radial growth measurements could be combined with other datasets to enhance further studies of tree growth in the Northern Rocky Mountains.
● Measurements of radial growth across environmental gradients are particularly useful for determining how climate impacts this generalist species.

## 1 Data

Here, we present measurements of raw radial growth, distance to pith, and calculated basal area increments (BAI) from 444 5-mm increment cores (237 trees) collected in July 2016 from the Beaverhead-Deerlodge National Forest, MT. Plot locations where increment cores were taken as well as code to calculate BAI are also included. Cores were collected from trees that were killed during a recent bark beetle outbreak (220 cores; 117 trees) as well as trees that survived the outbreak (210 cores; 113 trees) in twelve stands spanning north and south aspects and three elevational bands along a 600-m gradient. 14 additional cores were collected from 7 strip-attacked trees.

## 2 Experimental design, materials, and methods

### 2.1 Study area

Our study sites are located within the Boulder Mountains of the Beaverhead-Deerlodge National Forest, where elevation ranges from ~1400 m to ~3100 m. Study sites are located between ~1800 m and ~2500 m elevation. The area experienced a severe mountain pine beetle outbreak in the mid-2000s. Primary tree species in the area are lodgepole pine (*Pinus contorta*), Douglas-fir (*Pseudotsuga menziesii* (Mirb.) Franco), subalpine fir (*Abies lasiocarpa* (Hook.) Nutt), and whitebark pine (*Pinus albicaulis* Engelm.). According to the nearest climate station, located ~34 km away in Boulder, MT, January was the coldest month between 1880 and 2016, with an average minimum air temperature of −12.4 °C. July was the warmest month with an average maximum air temperature of 28.2 °C [5]. Within this period, annual precipitation averaged 279 mm, with most precipitation falling in June. The actual study site air temperature is likely lower and precipitation higher as Boulder, MT is located just outside the forested area at a lower elevation (1521 m).

### 2.2 Plot selection

Twelve plots were selected for the study from the Thunderbolt Creek and Boulder River drainages. The plots span both north and south aspects, and three elevational bands across a 600-m gradient. Potential plot locations were selected based on apparent lodgepole pine dominance, significant mortality due to mountain pine beetle, and stand access [6], [7]. Actual plots were selected upon visiting the sites, with selection determined by (1) dominance of lodgepole pine in the canopy, (2) substantial mountain pine beetle-caused mortality in the stand (> 40%), and (3) survival of at least 10 trees in the plot and immediate vicinity. Two plots were chosen within each aspect-elevation combination. Plots were required to be a minimum of 100 m from one another so as to limit spatial autocorrelation.

### 2.3 Increment core sampling

Ten beetle-killed trees were selected within a 10-m radius circular plot, and two increment cores were taken at 1.37-m height on opposite sides of the tree, perpendicular to the slope. Beetle-killed trees were randomly selected across the plot to obtain an even distribution of samples. Ten surviving trees of similar diameter to the beetle-killed trees were selected and cored within the plot. If ten surviving trees were not found within the plot, additional surviving trees close to the boundary of the plot (i.e., within 1 m) were used. Non-random sampling of surviving trees was used in order to minimize the difference in ages between surviving and beetle-killed trees. A total of 482 tree cores were collected for the study, with 444 included in the analysis. Thirty-eight cores were discarded from the analysis due to poor correlations with the master chronologies (see Section 4.4).

### 2.4 Increment core preparation

Increment cores were prepared according to standard dendrochronological techniques [2]. Master chronologies for each aspect-elevation combination were created using approximately ten cores from surviving trees at each location. Cores were cross-dated, then scanned at 2400 dpi. Annual ring widths were measured using CooRecorder 7.7 [3], and final chronologies quantitatively validated in COFECHA [4].

Basal area increment (BAI), a measure of growth, was calculated using ring widths and estimated distance to pith with the dplR package [8] in R. Distance to pith was estimated based on growth and curvature of the earliest observed rings if the pith was not present in the core [9]. Converting annual ring widths to BAI overcomes the decrease in ring width that occurs as a function of increasing tree size [10].
